# Nasal foreign bodies: description of types and complications in 420 cases

**DOI:** 10.1016/S1808-8694(15)30028-8

**Published:** 2015-10-19

**Authors:** Ricardo Rodrigues Figueiredo, Andréia A. Azevedo, Arthur Octávio de Ávila Kós, Shiro Tomita

**Affiliations:** a MSc student in ENT, UFRJ; Residence at the HUCFF-UFRJ.; b Otolaryngologist.; c Former Professor of Otolaryngology - UFRJ.; d Professor of Otolaryngology - UFRJ. Souza Aguiar Municipal Hospital - Rio de Janeiro RJ.

**Keywords:** nose, nasal cavities, foreign bodies

## Abstract

Nasal cavities foreign bodies are common accidents in children, sometimes leading, in accordance with the literature, to complications such as epistaxis and bronchoaspiration. Diagnosis is often made with anterior rhinoscopy, but sometimes nasal fibroendoscopy and imaging may be useful.

**Aim:**

To evaluate 420 cases of nasal foreign bodies removed in ENT Service of Souza Aguiar Hospital, Rio de Janeiro, as related to sex, age, type of foreign body and complications.

**Materials and method:**

420 cases of nasal foreign bodies removed in the ENT service of Souza Aguiar Hospital between December 1992 and December 1998 were evaluated according to the parameters related above.

**Results:**

We found higher incidence between 0 and 4 years of age, and the most frequently found foreign bodies were foam fragments, plastic pieces of little toys, beans and paper fragments. Complications occurred in 9.05% of the cases, epistaxis and vestibulitis being the commonest.

**Conclusion:**

Nasal foreign bodies are especially found between the ages of 0 and 4 years. In our study, foam fragments and small plastic objects were the most frequent foreign bodies found. Complications were found in 9.05% of the cases, headed by epistaxis and nasal vestibulitis.

## INTRODUCTION

Foreign bodies are a rather common problem, specially in pediatric otolaryngology, being frequently followed by complications, some with significant severity^1^, ^2^, ^3^. The first years of a child's life represent a phase of exploration and interaction with the environment. When they start moving by their own means (crawling and walking), the child starts having access to a number of objects that have to be duly explored. This process encompasses, amongst other things, the placement of objects in orifices, such as the ears, nose and throat^3^. Parent's laxness and lack of attention, leaving small objects at the child's reach and not properly watching over them, much contributes to this high incidence of foreign bodies. Ears, noses and throats are the most exposed orifices, hence the high incidence of foreign bodies in them.

In adults we may have cases which were inflicted on purpose or accidentally, the former being rarely seen in the nasal cavities on patients without psychiatric disorders. The accidents are mostly caused by insect that penetrate the nasal cavities or, even more rare, shifting of foreign bodies from mouth and hypopharynx to the back of the nose.

Nasal cavity foreign bodies are among those with the richest symptoms. After a few days in the nasal cavity there is nasal mucous-purulent discharge and foul odor4,5. It is a classic axiom that these two symptoms, specially if unilateral in children, until proven contrary, are highly suggestive of foreign bodies. Nasal obstruction and epistaxis may also occur. Diagnosis is carried out through anterior rhinoscopy, which shows most foreign bodies^4^, ^5^. When in doubt, complementary methods may be used, such as:

• Pass a small catheter through the nasal cavities, to check their permeability. This approach may cause epistaxis and mucosal lacerations, besides facilitating the aspiration of foreign bodies. Therefore, it must be abolished.
• Side simple x-rays are good only for metal foreign bodies (Figure 1).

**Figure 1a:**
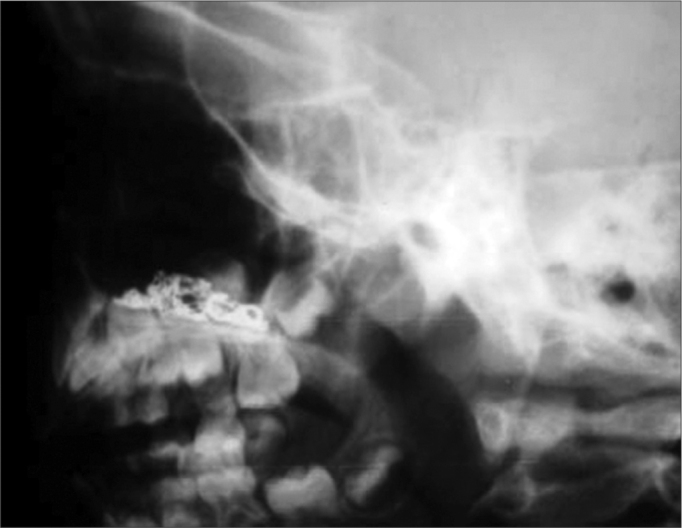
Simple fronto-nasal and side view x-rays.
• Nasal endoscopy, with the 2.7 rigid endoscope (ideal for children) or 4mm and 0 or 30° angle, or flexible nasopharyngoscopy. This is the best method for both the diagnosis and removal of foreign bodies in dubious cases. In some cases general anesthesia is required^6^, ^7^. Most nasal foreign bodies are located in the anterior portion of the nasal cavities, due to inferior turbinate extension (Figure 2)^6^. Such fact makes most nasal cavity foreign bodies of relative easy removal, in experienced hands. The ideal position for removal is having the patient sitting, on the lap of the person responsible, who will hold the child's arms and legs. A helper holds the head, which should be mildly extended (about 30°) (Figure 3). In some cases of difficult containment or a technically more challenging removal, general anesthesia may be necessary. In our experience we prefer the following instruments:

**Figure 2:**
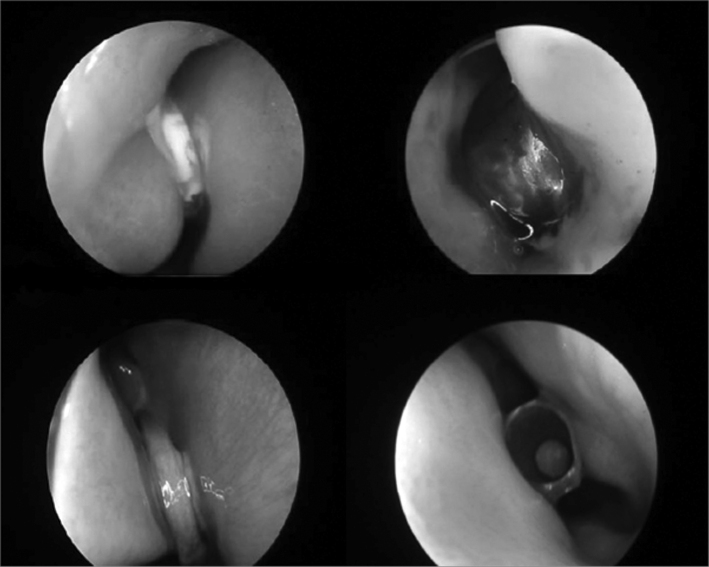
Nasal cavities foreign bodies (clockwise starting on the left upper corner): button, toy piece, pen lid, coin.

**Figure 3:**
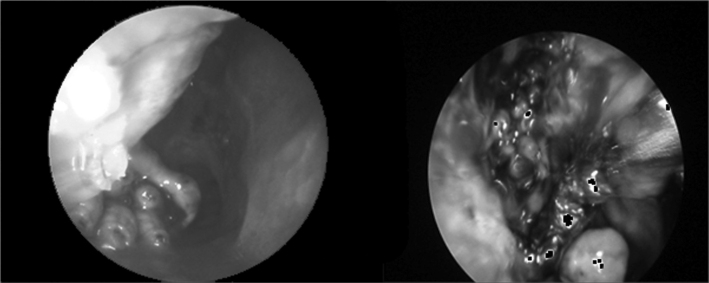
Nasal cavities myiasis.
• Blunt hooks
• Itard probes
• Bayonet forceps
• Hartmann-type forceps For hard foreign bodies, such as seeds and small plastic objects, we prefer the blunt hooks or the Itard probes.

For soft foreign bodies, such as sponge and paper fragments, we prefer the forceps, for simple capture and removal.

We recommend nasal flushing with saline solutions for 5 to 10 days afterwards. In some cases, diagnosis may not be so clear and the foreign body is not seen under simple anterior rhinoscopy. We may explore the cavity with blunt hooks; however, this may cause the aspiration of the foreign body. When in doubt, the ideal method is rigid or flexible nasal endoscopy. There are reports of removal with the help of the victim him/herself, through positive pressure and inflation through the mouth^10^. Live nasal cavity foreign bodies are more rare then their ear counterparts, with special attention to nasal cavity myiasis (Figure 3)^11^. This happens very frequently in street dwellers and alcoholics^11^. The larvae causes a suppuration and ample destruction of the nasal mucosa, leading to quick necrosis of septal cartilages and turbinates^11^, ^12^, ^13^. Its extension to the paranasal cavities and eye orbit is frequently witnessed. Treatment is done through extensive surgical debridment, preferably under general anesthesia, and broad-spectrum parenteral antibiotic agents^11^, ^12^ (we prefer the ceftriaxone/oxacilin combination, however cephalothin may also be employed, and also quinolones, such as gatifloxacin, parenterally). Our goal with this study is to assess the various parameters of a total of 420 cases of nasal cavity foreign bodies from the ENT-OS Department of the Souza Aguiar Hospital, and then trace an incidence profile of its occurrence in the city of Rio de Janeiro.

## MATERIALS AND METHODS

From December 1992 to December 1998 many nasal cavity foreign bodies data were noted, all of them removed by the authors in the ENT-OS Department of the Souza Aguiar Hospital, reference service for ENT foreign bodies in the State. The parameters noted were:

1. Gender
2. Age
3. Location – Right or left nasal cavities
4. Type of foreign body.
5. Complications, that may accrue from the placement of the foreign body, of its very presence or removal attempts.
6. Time passed between the foreign body placement and its removal. When it could not be accurately determined, it was called ignored. The data were analyzed in a purely descriptive fashion and the Ethics Committee of the Souza Aguiar Municipal Hospital approved this study.

## RESULTS

**Chart 1:**
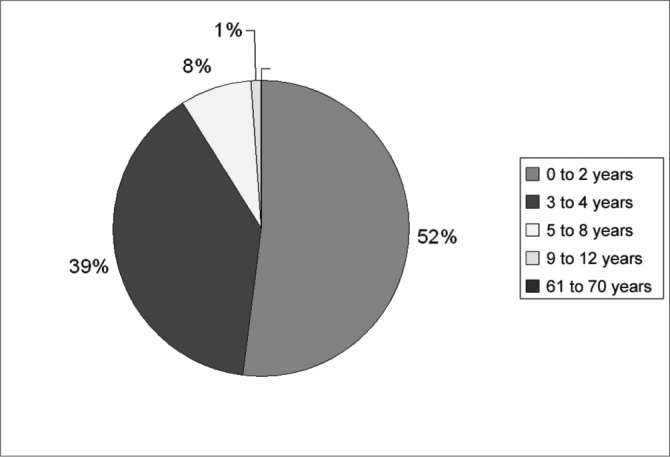
Age range distribution.

**Chart 2:**
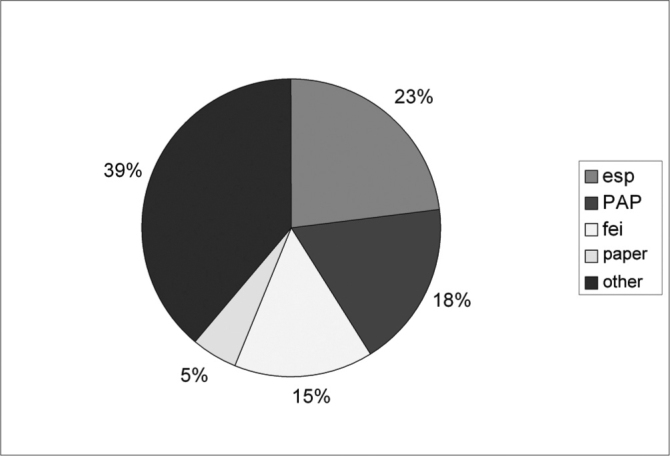
Types of nasal cavities foreign bodies.

The total number of cases was of 420, representing about 31% of all foreign bodies removed by our department. We observed 270 cases (64.29%) in the right nasal cavity and 150 cases (35.71%) in the left nasal cavity. As far as gender is concerned, we had 223 (53.09%) females and 197 (46.91%) males Chart 1 shows the distribution of cases according to age. As to the type of foreign body, of the 420 cases, the most commonly found were sponge fragments (96 cases, 22.86% from the total), small plastic objects, called PAP (76 cases, 18.09%), beans (62 cases, 14.76%) and paper fragments (23 cases, 5.47% from the total), as we can see on Chart 2. As far as complications were concerned, they were seen in 9.05% of the cases, epistaxis being the most common one (7.06%), followed by nasal vestibulitis (1.32%) and tissue necrosis (0.65%). The average time passed between the foreign body insertion and removal was of 9.71 hours. This time was unknown in 18.95% of the cases (neither the patient nor the people responsible for him/her knew exactly when the foreign body had been inserted).

## DISCUSSION

**Figure 4:**
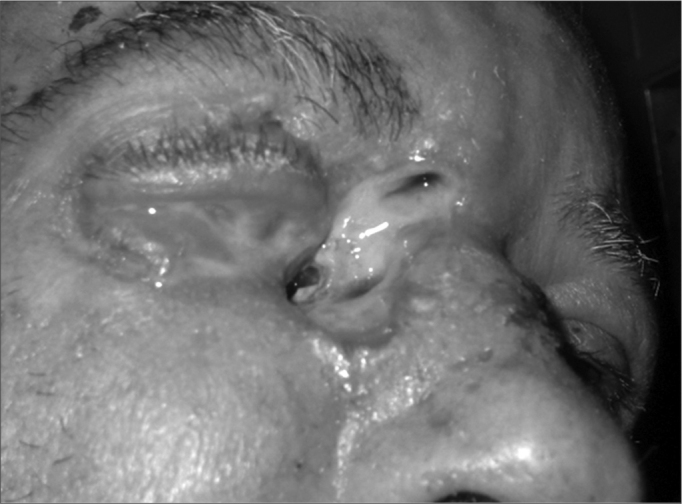
Nasal cavities myiasis with orbit complications.

The “Foreign Bodies” theme is one that concerns the whole society, fascinating all in its varieties, peculiarities and, eventually, bizarreness. Notwithstanding, we know that such accidents may lead to severe complications and, even death, although in this particular study we did not observe any fatal outcome. Otolaryngology deals with most of the natural body orifices through which foreign bodies may be introduced, namely the mouth, nostrils and ears. We have only the anus; urethra and vagina left, and because they are bodily parts usually covered by clothes, in most of our daily activities, they are more rarely used for this purpose. We saw a balance in gender distribution and a predominance of cases in the right nasal cavity (64.29% of the cases). We believe this finding is due to a greater occurrence of right-handed people in the general population. Analyzing the distribution by age range, there is a predominance of occurrences between 0 and 2 years (52.62%) and between 2 and 4 years (38.81%). We may then conclude that nasal cavity foreign bodies are more common in the first four years of life, when we observed 91.43% of the cases. We had only one adult case, a homeless man with nasal myiasis. Sponge fragments are the most commonly found foreign bodies in the nasal cavities (22.86%), being usually removed from pillows and damaged mattress. Therefore, bringing awareness to the parents in regards of this problem is of paramount importance. They quickly give off a foul smell between^24^ and 48 hours and are better removed with the Hartmann and Alligator forceps. Nasal flushing with saline solution should be prescribed after a foreign body removal. Now, the small plastic artifacts (PAP), which account for 18.09% of the cases, they usually come from toys with detachable parts or those easily breakable by children. Once again, parent education is fundamental, as well as educating the toy industry that should always tell the recommended age for each type of toy. It is important to remember those toys bought from street vendors with inadequate or inexistent inspection; they usually do not follow these guidelines. These foreign bodies may be removed with the aid of blunt hooks or forceps, depending on their shape and location within the nasal cavity. Beans that in general are the most frequently found foreign bodies in Otolaryngology, account for 14.76% of the cases. Usually bean grains, as it happens to other common edible seeds, fall on the kitchen floor during cooking and are found by children. However, we had cases of mothers would gave their children beans to play with. They are easily removed with blunt hooks. Paper fragments were found in 5.47% of the cases, also giving off foul odor, although not as bad as the ones generated by sponges. They are easily removed with forceps. Different types of foreign bodies were found, some very curious indeed, such as meat pieces and cell phone buttons. Different types of seeds such as corn, peas and orange seeds – which are also very common. Mothballs, so frequent in the past, are rarely found today. Special attention should be paid to cases of nasal myiasis, which brings about greater morbidity with a high complication rate, including ample destruction of the turbinates and nasal septum, besides orbit complications and even neurological complications^11^, ^12^, ^13^ (Figure 4). The patient we had in this study underwent surgical debridment and parenteral antibiotic therapy, having a broad septal perforation as sequelae. Systemic use of Ivermectin – antiparasitic agent – may be considered^12^. The complication rate was relatively low (9.05%). However, it is important to stress that Souza Aguiar Hospital is a referral center for foreign bodies, its professionals are widely experienced in this topic. Although the data from this study do not prove this fact, in our observations the complication rate increases as the patients are seen by less experienced professionals in foreign body management, and this reinforces the need for training the otolaryngology resident in ER services. It is important to educate clinicians and pediatricians about the need to have proper instruments and techniques in order to attempt removal of foreign bodies. Still today it is not rare to see patients with nasal foul odor caused by foreign bodies being treated by antibiotics for sinusitis, which is unacceptable. Often times we may risk a nasal cavity foreign body diagnosis just by the odor given off by the patient as he/she enters our office, and it is rare that sinusitis would generate such foul odor. Epistaxis usually happens during foreign body removal, given the frailty of the nasal mucosa, damaged even further by the secondary inflammatory process^6^, ^9^. The parents or guardians must be educated and previously tranquilized as to this possibility. Epistaxis is, usually, mild and of quick resolution. In our study, no patient required cauterizing or nasal packing, because nasal bleeding was controlled by finger compression only.

**Figure 1b:**
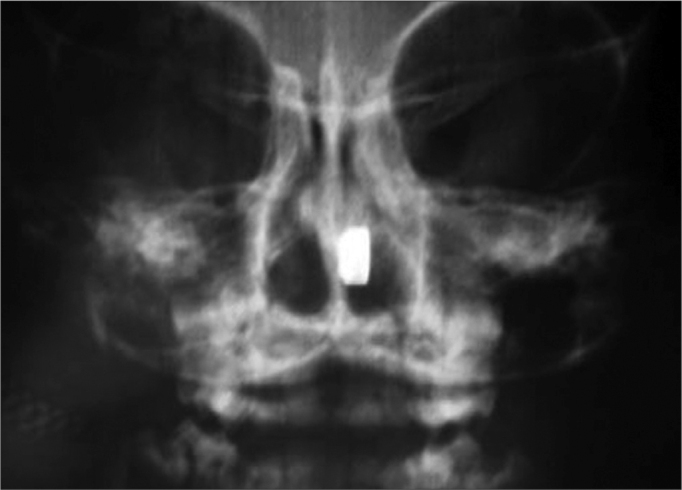
Simple fronto-nasal and side view x-rays.

Some cases of nasal vestibulitis secondary to foreign bodies may require parenteral antibiotic therapy, preferable cephalexin together with saline solution flushing of the nasal cavities.

## CONCLUSION

Nasal cavities foreign bodies are accidents mostly seen in children up to 4 years of age, in most situations they are avoidable if the parents are careful and attentive. Our data may as well reflect the whole scenario in the city of Rio de Janeiro because the Souza Aguiar Hospital is a referral center for foreign body management. Sponge fragments, small plastic pieces and beans were among the most found foreign bodies. Although in most situations they are of relative simple resolution, there may be complications such as epistaxis and vestibulitis. It is worth highlighting that, although we did not see any occurrence in our study, every nasal cavity foreign body may be aspirated, thus becoming a potential bronchi foreign body. It is of paramount importance to train the otolaryngology resident in ER settings, because as our experience has proven, the complication rate is directly related to the lack of experience or proper instruments.

## THANKS

To all otolaryngologists and nurse assistants of the Souza Aguiar Municipal Hospital.
